# Hyperpolarized Amino Acid Derivatives as Multivalent Magnetic Resonance pH Sensor Molecules

**DOI:** 10.3390/s18020600

**Published:** 2018-02-15

**Authors:** Christian Hundshammer, Stephan Düwel, David Ruseckas, Geoffrey Topping, Piotr Dzien, Christoph Müller, Benedikt Feuerecker, Jan B. Hövener, Axel Haase, Markus Schwaiger, Steffen J. Glaser, Franz Schilling

**Affiliations:** 1 Department of Nuclear Medicine, Klinikum Rechts der Isar, Technical University of Munich, 81675 München, Germany; christian.hundshammer@tum.de (C.H.); stephan.duewel@tum.de (S.D.); geoff.topping@lrz.tu-muenchen.de (G.T.); realtoughmonkey1980@gmail.com (P.D.); benedikt.feuerecker@mytum.de (B.F.); markus.schwaiger@tum.de (M.S.); 2 Department of Chemistry, Technical University of Munich, 85748 Garching, Germany; david.ruseckas@mnet-online.de (D.R.); glaser@tum.de (S.J.G.); 3 Munich School of Bioengineering, Technical University of Munich, 85748 Garching, Germany; axel.haase@tum.de; 4 Department of Radiology, Medical Physics, University Medical Center Freiburg, Faculty of Medicine, University of Freiburg, 79106 Freiburg, Germany; christoph.mueller.rdiag@uniklinik-freiburg.de; 5 German Consortium for Cancer Research (DKTK), 69120 Heidelberg, Germany; 6 German Cancer Research Center (DKFZ), 69120 Heidelberg, Germany; 7 Section for Biomedical Imaging, Molecular Imaging North Competence Center (MOINCC), Department for Radiology and Neuroradiology, University Medical Center Kiel, University Kiel, 24118 Kiel, Germany; jan.hoevener@rad.uni-kiel.de

**Keywords:** pH sensors, hyperpolarized, dissolution dynamic nuclear polarization, magnetic resonance spectroscopic imaging, nuclear magnetic resonance, amino acids

## Abstract

pH is a tightly regulated physiological parameter that is often altered in diseased states like cancer. The development of biosensors that can be used to non-invasively image pH with hyperpolarized (HP) magnetic resonance spectroscopic imaging has therefore recently gained tremendous interest. However, most of the known HP-sensors have only individually and not comprehensively been analyzed for their biocompatibility, their pH sensitivity under physiological conditions, and the effects of chemical derivatization on their logarithmic acid dissociation constant (p*K*_a_). Proteinogenic amino acids are biocompatible, can be hyperpolarized and have at least two pH sensitive moieties. However, they do not exhibit a pH sensitivity in the physiologically relevant pH range. Here, we developed a systematic approach to tailor the p*K*_a_ of molecules using modifications of carbon chain length and derivatization rendering these molecules interesting for pH biosensing. Notably, we identified several derivatives such as [1-^13^C]serine amide and [1-^13^C]-2,3-diaminopropionic acid as novel pH sensors. They bear several spin-1/2 nuclei (^13^C, ^15^N, ^31^P) with high sensitivity up to 4.8 ppm/pH and we show that ^13^C spins can be hyperpolarized with dissolution dynamic polarization (DNP). Our findings elucidate the molecular mechanisms of chemical shift pH sensors that might help to design tailored probes for specific pH in vivo imaging applications.

## 1. Introduction

The pH is an important physiological parameter that is tightly regulated in living organisms by intrinsic buffer systems. Several diseases such as inflammation, ischemia and cancer are associated with metabolic changes affecting the extracellular tissue pH [1,2,3,4,5,6]. Biocompatible sensor molecules that can be used to non-invasively measure pH in vitro and in vivo are therefore highly valuable to study disease induced metabolic changes. In the clinic, measuring pH would be helpful for diagnosis of malignant tissues, monitor response to treatment, and tailor therapies to patient-specific phenotypes [7,8,9]. 

In the past, several pH-sensitive small molecules and nanoparticles for positron emission tomography, fluorescence, and optoacoustics have been proposed [10]. In contrast to these methods, magnetic resonance imaging (MRI) approaches do not rely on ionizing radiation, offer a high penetration depth and excellent soft tissue contrast, and allow fast acquisition of high-resolution anatomical images at the same time. Saturation transfer between bulk water and exchangeable protons of molecules with pH-dependent exchange rates [11] or relaxivities of lanthanide complexes can be used for pH imaging [12]. Additionally, thermally polarized signals of molecules bearing pH sensitive ^1^H, ^19^F [13] and ^31^P [14] nuclei can be exploited for in vivo pH measurements. IEPA ((+/−)2-imidazole-1-yl-3-ethoxycarbonylpropionic acid) [15,16] and ISUCA ([(+/−)2-(imidazol-1-yl)succinic acid]) [17] were applied for proton pH imaging in preclinical studies, while histidine was able to measure pH with spatial localization in human brain after oral loading of the amino acid [18]. 

During the last two decades, efficient hyperpolarization (HP) techniques such as spin exchange optical pumping (SEOP) [19], parahydrogen induced polarization (PHIP) [20,21,22] and dissolution dynamic nuclear polarization (DNP) have been developed to increase the thermal NMR signal by more than five orders of magnitude and to overcome signal limitations of classical NMR and MRSI (magnetic resonance spectroscopic imaging) [23]. pH-sensitive molecules with spin-1/2 nuclei (^13^C [3,24,25,26], ^15^N [27,28], ^31^P [29], ^89^Y [30] and ^129^Xe [31,32]) have been polarized with these methods and were applied in vitro, while only ^13^C-labelled sensors have been used in vivo so far [3,12,29]. ^13^C-nuclei can be polarized to high levels of above 70%, they have a fairly high gyromagnetic ratio and a relatively long *T*_1_ especially for carbonyls, quaternary or deuterated carbons [33]. Nevertheless, almost all of the presented molecules were only characterized individually and a generally applicable strategy for the design of biocompatible magnetic resonance pH sensors is needed. 

Proteinogenic amino acids are essential for living organisms. They are used as precursors for neurotransmitters, nucleotides, co-factors, and proteins and are crucial metabolic carriers or serve as energy sources in phases of low nutrient supply. In this work, we systematically analyzed the effects of derivatization on the p*K*_a_ of natural amino acids. We characterized the NMR pH sensitivity of several spin-1/2 nuclei with high natural abundance (^31^P) or that can be isotopically enriched (^13^C, ^15^N) in amino acid derivatives. Furthermore, we investigated the potential of HP using dissolution dynamic nuclear polarization (DNP) for the most interesting candidates to ultimately obtain novel in vivo pH biosensors. 

## 2. Materials and Methods

### 2.1. Chemicals

Non-labelled and labelled chemicals were purchased from Sigma Aldrich (Taufkirchen, Germany) and Cambridge Isotope laboratories (Andover, MA, USA), respectively.

### 2.2. Synthesis of ^15^N-Labelled 2,3-Diamino Propionic Acid (DAP)

Synthesis of DAP was performed as described previously [34,35]. [1,4-^13^C_2_]aspartic acid or [1,4-^13^C_2−_2-^15^N]aspartic acid (1 equivalent) was dissolved in fuming sulfuric acid (30%) under ice-bath cooling followed by addition of dry chloroform. Sodium azide (2 equivalents) was then added in small portions under reflux for 5 h. After that, the mixture was brought to room temperature and stirred for another 2 h under reflux. After ice-bath-cooling, the chloroform layer was removed, and ice was added to dissolve the paste-like residue. The solution was passed through a DOWEX-column (H^+^-form, 100–200 mesh) that was washed with 0.5 N HCl followed by washing with water to neutrality. Elution of the raw product was performed with 4 N NH_3_ in MeOH. ninhydrin-positive fractions were pooled and concentrated by rotary evaporation. The final addition of 6 N HCl precipitated diaminopropionic acid hydrochloride at pH ~ 2. 

The raw product was filtered, washed with methanol and acetone and recrystallized twice in methanol yielding white crystalline [1-^13^C, 2-^15^N]2,3-diaminopropionic acid hydrochloride (^13^C-^15^N-DAP or [1-^13^C, 2,3-^15^N_2_]2,3-diaminopropionic acid hydrochloride (^13^C-^15^N_2_-DAP). The experimental yield for both was about 40%. 

### 2.3. Synthesis of Serine Ethyl and Propyl Ester

Thionyl chloride (1 equivalent, 36 mmol) was added slowly to dry absolute ethanol or propanol (47 mL and 13 mL) cooled to 0 °C. After the addition of serine (1 equivalent, 10 mmol) and removal from the ice-bath, the mixture was stirred for another two minutes before slowly being heated to reflux temperature, dissolving serine. After stirring for ten minutes, the mixture was cooled to 0 °C, followed by the addition of dry tert-butylether (150 mL) leading to crystallization of serine ethylester crystals. The crystals were filtered and washed with tert-butylether, yielding serine ethylester in form of white crystals (experimental yield: 49% and 16% for serine ethyl and propyl ester, respectively).

### 2.4. NMR Measurements

Carbon NMR spectra were acquired either on a 300 MHz or 600 MHz NMR spectrometer (Avance III, Bruker BioSpin, Billerica, MA, USA) in 5 mm NMR tubes and at 25 °C if not stated differently. Carbon spectra were measured using ^1^H-decoupling, phosphorus and nitrogen without decoupling. For the determination of the pH-dependent chemical shifts, amino acids and ^13^C-urea were dissolved in 1 M KCl in 90% H_2_O/10% D_2_O and adjusted with 10 M HCl and 10 M KOH to the desired pH values measured with a standard glass electrode (pH meter: ProLab 4000, SI Analytics or Lab850, Schott, pH electrode: N6000A, Weilheim, Germany). If not stated differently, final concentrations of non-labelled and labelled compounds were 250 mM and 2.5 mM, respectively. 

For analysis of the temperature dependency, solutions were measured at 25 °C, 37 °C and 50 °C controlled by an air heating within the NMR spectrometer. DAP and serine amide (SA) concentrations were varied for analysis of the dependency of the sensor concentration on the chemical shift (100 mM, 250 mM, 500 mM). In cases where effects of the ionic strength on the NMR sensitivity was tested, respective amounts of KCl were added, taking the sensor concentration into account. Then, 0.4 and 2.0 equivalents (for SA, 0.4 and 2.5 equivalents for DAP) of CaCl_2_ were added to solutions containing 250 mM of amino acid to test for potential interaction or complexation of SA/DAP with bivalent metal ions. Chemical shift sensitivity in presence of proteins was tested with increasing bovine serum albumin (BSA) concentrations (0, 50, 75, 100 and 210 g/L) at fixed pH values (pH 6.6, 7.0, and 7.4), which were adjusted after each addition of BSA. 

Carbon and nitrogen NMR spectra of 300 mM solutions of ^13^C-^15^N-DAP and ^13^C-^15^N_2_-DAP, carbon spectra of a 100 mM solution of ^13^C-SA, and phosphorus spectra of a 300 mM solution of P-Ser were acquired on a 300 MHz spectrometer (all in solutions of 90% H_2_O/10% D_2_O). [^13^C]urea was added to the solutions as internal chemical shift reference for ^13^C-measurements.

### 2.5. Analysis of NMR Titration Curves

NMR spectra were analyzed with MestReNova 10.0 (Mestrelab Research, Santiago de Compostela, Spain). 5 Hz line broadening using an exponential filter was applied to all NMR spectra. The resonance signal of urea was set to 165 ppm and used as an internal reference for carbon spectra. Nitrogen and phosphorus spectra were referenced to the respective spectrometer frequencies. 

The pH-dependent chemical shifts were fitted to the following Equation (1) [36]:

(1) $$\delta_{obs}=\frac{\delta_{min}+\sum_{i=1}^{n}\delta_{max,i}\;10^{\left(\sum_{j=n-i+1}^{n}pK_{a,j}\right)-ipH}}{1+\sum_{i=1}^{n}10^{\left(\sum_{l=n-i+1}^{n}pK_{a,l}\right)-ipH}}$$

*δ_obs_* is the measured chemical shift, *δ_min_* the lowest observable chemical shift, n is the number of proton exchange sites, *δ_max_* is the highest observable chemical shift for the respective deprotonated species, p*K*_a_ is the logarithmic acid dissociation constant of the respective nucleus, and the pH value was measured with a glass electrode. The pH sensitivity Δ*δ* [ppm] is given as the chemical shift difference between pH 6.4 and pH 7.6 calculated from fit curves. 

### 2.6. WST-1 Cytotoxicity Tests

T-cell murine lymphoma (EL4)-derived cells were cultivated in RPMI 1640 medium (high glucose, ATCC, Manassas, WV, USA). Increasing amounts of serine amide hydrochloride or 2,3-diaminopropionic acid hydrochloride from stock solutions in PBS, pH 7.1 and 10 µL dimethyl sulfoxide (DMSO) were added to 10 mL culture medium containing 5.5–6.1 × 10^5^ cells (final amino acid concentrations: 0, 1.25, 2.5, 5, 10, and 20 mM). As positive control, 10 µL of 15 mM etoposide solution in DMSO was added to a separate 10 mL cell culture. Flasks were incubated for 22 h at 37 °C and 5% CO_2_. Cell media were then changed, and a 100 µL sample of each culture was taken for a water-soluble tetrazolium 1 (WST-1) assay. Each 100 µL cell culture sample was incubated with 10 µL of the WST-1 reagent for 2 h at 37 °C and 5% CO_2_, after which the optical absorbance at 450 nm was measured using an Infinite 200 PRO (Tecan, Männedorf, Switzerland) absorbance microplate reader. The WST1 assay was carried out three times for each condition.

### 2.7. ^13^C-Hyperpolarization and T_1_ Measurements 

[1-^13^C]serine amide hydrochloride (SA), [1-^13^C]2,3-diaminopropionic acid hydrochloride (DAP) and pyruvate (PA) were prepared for polarization (1–3 h, Hypersense, Oxford Instruments, Abingdon, UK) according to Table 1. 

Solid build up curves were normalized to the respective ^13^C molar amount (molar solid build up in units per mole; u.*mol^−1^) and fitted to an exponential function (Equation (2)).

(2) $$P_{molar\;solid\;build\;up}\left(t\right)=P_{solid\;state,max}\cdot \left(1-e^{\left(t\cdot T^{-1}\right)}\right)\cdot n$$

With *P_molar solid build up_*(*t*) being the solid state polarization at time *t*, *P_solid state,max_* being the maximum achievable solid state polarization, *T* being the solid state build up constant and *n* being the molar amount of the respective polarized molecule. 

The samples were dissolved in pressurized (10 bar) and heated (180 °C) 80 mM Tris (Tris(hydroxymethyl)-aminomethan) buffered solution, which was adjusted with KOH or HCl to reach distinct pH values. Final concentrations of SA, DAP and PA were: 28.5 ± <0.1 mM (pH 5.3 ± 0.8, *n* = 3), 20.9 ± 1.7 mM (pH 5.8 ± 0.4, *n* = 3), and 103.3 ± <0.1 mM (pH 7.2 ± 0.2, *n* = 3). For SA and DAP, ~100 mM vitamin C were added to the dissolution buffer to quench the free radical and also decreased the pH of the solution to acidic pH. ^13^C *T*_1_ measurements of hyperpolarized substances were performed in 5 mm standard NMR tubes on a 1 T NMR spectrometer (Spinsolve Carbon, Magritek, Aachen, Germany) using the following acquisition parameters: 4° flip angle and 3 s repetition time (TR) for PA; 1° flip angle, TR = 3 s or 20° flip angle TR = 5 s for SA and DAP. A 25 Hz line broadening using an exponential filter was applied to all NMR spectra of hyperpolarized compounds. Hyperpolarized signals (*S_hyper,obs._*) at time point *t* were flip angle corrected according to Equation (3).

(3) $$S_{hyper,corr.}\left(t\right)=\frac{S_{hyper,obs.}}{\cos{\left(\alpha \right)}^{n-1}}$$

with *S_hyper,corr._*(*t*) being the flip angle corrected hyperpolarized signal at time *t*, α the nominal flip angle, and *n* the indexed number of each individual experiment. Flip angle corrected *T*_1_ decay curves were fitted to a monoexponential decay curve (Equation (4)) and interpolated to the time-point of dissolution (*S_hyper_* (*t* = 0).

(4) $$S_{hyper,corr.}\left(t\right)=S_{hyper}\left(t=0\right)\cdot e^{-t\cdot T_{1}^{-1}}$$

with *T*_1_ being the spin lattice relaxation time. 

Hyperpolarized signal enhancements (*ε*) and liquid state polarization levels (*P_hyper_*) were calculated using Equation (5).

(5) $$P_{hyper}=\frac{S_{hyper}\left(t=0\right)\cdot \sin\left(\alpha \right)}{S_{thermal}\cdot \sin\left(90{}^{\circ}\right)}\cdot \tanh\left(\frac{\gamma \hbar B_{0}}{2k_{B}T}\right)=\varepsilon \cdot P_{thermal}$$

with *γ* being the gyromagnetic ratio of carbon, ħ the reduced Planck constant, *k*_B_ the Boltzmann constant, and *T* = 300.15 K (constant spectrometer temperature). *S_thermal_* is the thermal signal of a 10 M ^13^C-urea solution acquired at the same spectrometer with a single shot (flip angle 90°) and corrected for the concentration of the respective hyperpolarized molecule as given in Equation (6).

(6) $$S_{thermal}=\frac{signal_{10Murea,\;90{}^{\circ}}\cdot c\left(HP\;molecule\right)}{10\;\mathrm{mol}\cdot L^{-1}}$$

### 2.8. Thermal Phantom Imaging and Back-Calculation of pH Maps

Custom-made 3D-printed phantoms with letter-shaped compartments were used to acquire spatially resolved pH maps (coronal orientation) from the thermal signals of 250 mM ^13^C-SA (phantom 1: “TUM MRI”) and ^13^C-DAP (phantom 2: “pH = pK_a_ + log(A^−^/HA)”) in 100 mM citric acid/200 mM disodium phosphate buffer (universal buffer) supplied with 250 mM ^13^C-urea as internal reference and 2 mM gadolinium chelate (Dotarem, Guerbet, Villepinte, France). Distinct pH values were adjusted with 10 M KOH or 12 M HCl. 

MR images were acquired with a 7 T magnet operating with Bruker AVANCE III HD electronics (Bruker Biospin, Billerica, MA, USA). A dual-tuned ^1^H/^13^C volume coil (RAPID Biomedical, Rimpar, Germany) with inner diameter 31 mm was used for radiofrequency (RF) transmission and signal reception. Proton localization images were acquired with a fast low angle shot (FLASH) sequence using the following acquisition parameters: repetition time 150 ms, echo time 3.1 ms, flip angle 15°, matrix size 380 × 260 (phantom 1), 420 × 260 (phantom 2), field of view (FOV) 38 mm × 26 mm (phantom 1) or 42 mm × 26 mm (phantom 2), slice thickness 5 mm (phantom 1) or 3mm (phantom 2), four averages, total scan time 2 min 36 s, receiver bandwidth 100 kHz and excitation bandwidth 12 kHz. ^13^C spectroscopic images were acquired with a 2D phase-encoded chemical shift imaging sequence (CSI), with repetition time 300 ms, nominal flip angle 30°, matrix size 72 × 52, (phantom 1) and 84 × 52 (phantom 2), field of view and slice thickness matching the proton localization images, 12 (phantom 1) or 14 (phantom 2) averages for each of six (phantom 1) or four (phantom 2) repetitions, total scan time 23 h 43 min (phantom 1) or 20 h 23 min (phantom 2), receiver bandwidth 4 kHz, 256 points acquired per phase-encode, spectral resolution 7.8 Hz, excitation bandwidth 12 kHz. Automated iterative linear shimming on the proton signal was run prior to proton acquisition. Reference power was determined automatically for protons by the scanner and used a predetermined value for ^13^C based on separate phantom measurements. ^13^C images were reconstructed in Matlab (Mathworks, Natick, MA, USA). In the spectral dimension, 15 Hz line broadening was applied before Fourier transformation. 

NMR signals were summed over all repetitions. For each voxel, the maximum intensity of each peak was determined automatically with Matlab (Mathworks, Natick, MA, USA) and the respective peak frequencies were used to determine the chemical shift difference between the resonance signal of urea set to 165 ppm and the respective sensor signal. The pH maps were back-calculated (numerical for DAP and analytical for SA) using the mean p*K*_a_ and chemical shift values obtained from the three respective pH titration curves of DAP and SA at different temperatures acquired with NMR spectrometers and with the fit parameters in Table 2. 

## 3. Results

**Figure 1:**
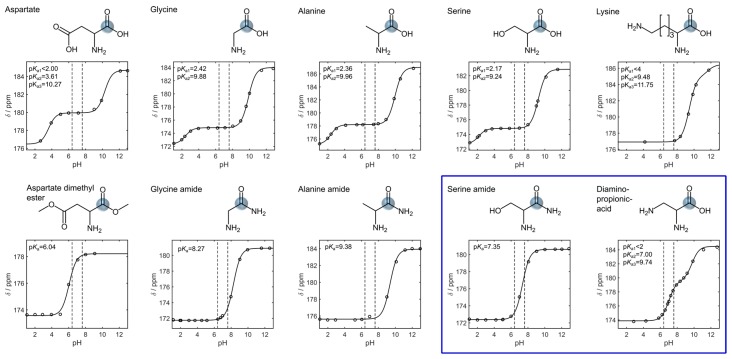
pH-dependent chemical shift of carbonyl ^13^C-atoms of representative acidic, hydrophobic, neutral, polar, and basic canonical amino acids (from left to right, top row) and one respective derivative each (bottom row). The p*K*_a_ of the carboxylic acid and the amino group of naturally occurring amino acids are below pH 3 and above pH 9, respectively. Side chains as shown for aspartate may have a higher (lower) p*K*_a_, but none of them is in a range relevant for in vivo pH imaging. Around their p*K*_a_, carbonyl carbons of canonical amino acids exhibit a pH dependent chemical shift (top row). Esterification, amide formation, and carbon chain length reduction lower the p*K*_a_ of amino acids toward the relevant range for in vivo pH imaging yielding derivatives such as SA and DAP (blue box), which show a strong chemical shift between pH 6.4 and pH 7.6 (bottom row). The pH range relevant for in vivo pH imaging is indicated with vertical dashed lines in each pH nuclear magnetic resonance (NMR) titration plot. NMR pH titrations curves were obtained from 250 mM amino acid solutions in 1 M KCl containing 10% D_2_O. Spectra were reference to the resonance signal of 2.5 mM ^13^C-urea set to 165 ppm.

Amino acids bear carbonyl carbons with a relatively long *T*_1_. They are amenable for hyperpolarization [37,38,39] and exhibit a pH-dependent chemical shift around the p*K*_a_ of their pH sensitive moieties (Figure 1 and Figure S1): the amino group (p*K*_a_ < 9), the carboxylic acid group (p*K*_a_ < 3), and in some cases a side chain group that has a p*K*_a_ close to physiological pH like histidine (p*K*_a2_ = 6) and cysteine (p*K*_a2_ = 8.1). 

**Figure 2:**
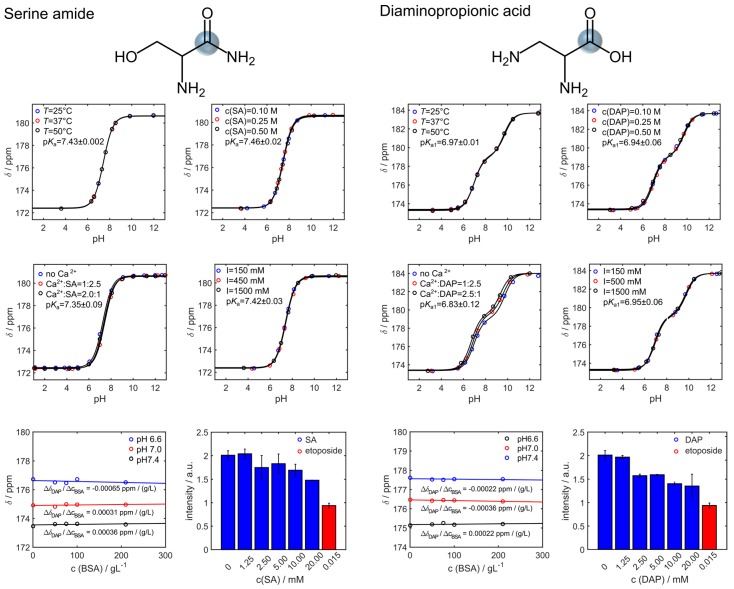
Dependency of pH sensitivity of serine amide (SA) and 2,3-diaminopropionic acid (DAP) under varying environmental conditions mimicking different physiological parameters. The ^13^C chemical shift of SA (left two rows) and DAP (right two rows) are rather independent of temperature, sensor concentration, ionic strength, and the presence of proteins (BSA). DAP appears to slightly interact with Ca^2+^ ions, which was not observed for SA. Water-soluble tetrazolium (WST) cytotoxicity tests with EL4 tumor cells showed a slightly reduced metabolic activity at concentrations above 1.25 mM of DAP. At concentrations above 10 mM, a decrease in metabolic activity is observable for both DAP and SA, which is however not as prominent as for the positive control (etoposide). Note that the concentration of etoposide is 2 to 3 magnitudes lower than the one of DAP and SA. NMR pH titrations curves were obtained from 250 mM amino acid solutions except for experiments analyzing the effect of the sensor concentration on the pH-dependent chemical shifts. In that case, measured concentrations were 100 mM, 250 mM and 500 mM. Spectra were referenced to the resonance signal of 2.5 mM ^13^C-urea set to 165 ppm.

In order to exploit the pH sensitivity of these moieties for hyperpolarized in vivo pH imaging, their p*K*_a_ needs to be adjusted to be in a range that is at the physiological pH (pH 7.0 ± 0.6 [12]) or rather acidic [1,3,4,5,6]. The p*K*_a_ of histidine (His) and cysteine (Cys) are close to that range but their carbonyl chemical shift sensitivity is rather low (histidine: Δδ = 0.7 ppm, cysteine: Δδ = 0.2 ppm, for all other ^13^C shifts of histidine and cysteine see Figure S2). Esterification or amidation removes the carboxylic acid proton and the pH sensitivity of the carboxylic acid group’s carbon chemical shift, which leads to a lowering of the amino group’s p*K*_a_ by up to more than four pH units (Figure 1 and Figure S1). For instance, aspartate has a p*K*_a2_ = 10.27, which is lowered to p*K*_a_ = 6.04 by esterification of both carboxylic acid groups. Glycine methyl ester has a p*K_a_* = 7.52 that is two pH steps below the one of glycine (p*K*_a_ = 9.88). Serine amide (SA) has a p*K*_a_ = 7.35 and its carbonyl carbon shows a strong pH shift of Δδ = 4.2 ppm in the physiological pH range. A decreasing number of carbon atoms in the ester alkyl chain has negligible effects on the p*K*_a_ as observed for serine propyl (*pK*_a_ = 7.74, Δδ = 4.2 ppm), serine ethyl (p*K*_a_ =7.47, Δδ = 4.5 ppm), and serine methyl ester (p*K*_a_ = 7.32, Δδ = 3.8 ppm, see Figure S1). Shortening of a basic amino acid’s chain length decreases the p*K*_a_ as measured for lysine (p*K*_a2_ = 9.48) > ornithine (p*K*_a2_ = 9.19) > 2,4-diaminobutyrate (p*K*_a_ = 8.38) > 2,3-diaminopropionic acid (DAP) (p*K*_a_ = 7.00). Notably, DAP has the same pH sensitivity (Δδ = 4.2 ppm) in the physiological pH range as SA. Although amino acid alkyl esters also exhibit a similarly high pH shift around this range, they hydrolyse in solution, forming the respective canonical amino acids and alcohols (Figure S3). In contrast, DAP and amino acid amides like SA are stable in aqueous solution. We therefore tested if their pH-dependent chemical shift is influenced by parameters that vary under in vivo conditions and that could thus affect the pH determination (Figure 2). 

Their chemical shift is not decisively altered or impeded by temperature, sensor concentration, ionic strength, or in presence of proteins. DAP appears to weakly interact with bivalent metal ions such as calcium, which was not observed for SA. WST cytotoxicity tests with EL4 tumor cells show a slightly reduced metabolic activity at concentrations above 1.25 mM of DAP. This is not observed for SA at concentrations relevant for in vivo applications (<10 mM).

**Figure 3:**
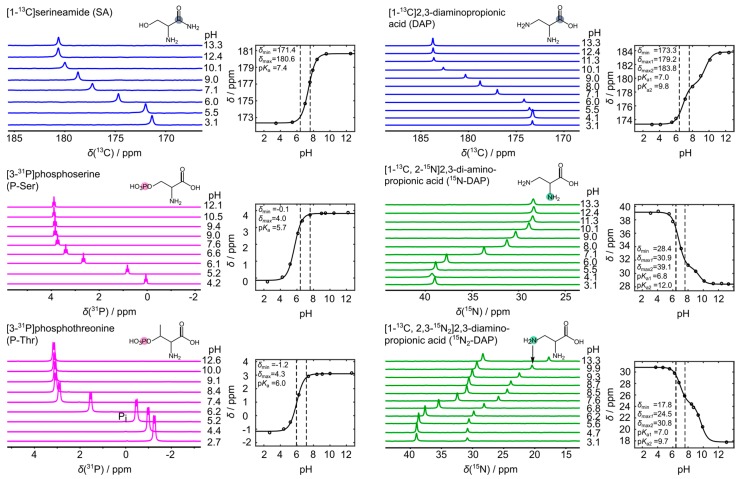
NMR titration curves of P-Ser, P-Thr, ^13^C-SA, ^13^C-DAP, ^13^C-^15^N-DAP, and ^13^C-^15^N_2_-DAP. ^13^C (blue), ^31^P (purple), and ^15^N (green) NMR pH titration series are shown for the respective amino acid given with their chemical structure. Measured nuclei are marked with filled circles of their respective colors. Fit curves and fitting parameters for pH-dependent chemical shifts are given next to each NMR series. Dashed lines mark the range pH 6.4–7.6. Concentrations of P-Ser, P-Thr, ^13^C-DAP, ^13^C-^15^N-DAP, and ^13^C-^15^N_2_-DAP were 300 mM. For, ^13^C-SA, a 100 mM solution was measured. ^13^C spectra were referenced to the resonance signal of 250 mM ^13^C-urea set to 165 ppm. ^15^N and ^31^P spectra were reference to the respective spectrometer frequency.

Figure 3 demonstrates that spin-1/2 nuclei other than ^13^C exhibit a pH-sensitive chemical shift in the physiologically and pathologically relevant pH range. The nitrogen chemical shifts of ^15^N-labelled DAP are Δδ = 4.8 ppm (2-^15^N) and Δδ = 4.6 ppm (3-^15^N). Notably, we also observed pH-sensitive ^31^P chemical shifts of P-Ser and P-Thr in that range (Δδ = 1.2 ppm and Δδ = 1.8 ppm), which were not observed for the ^13^C-carbonyl (Figure S4).

**Figure 4:**
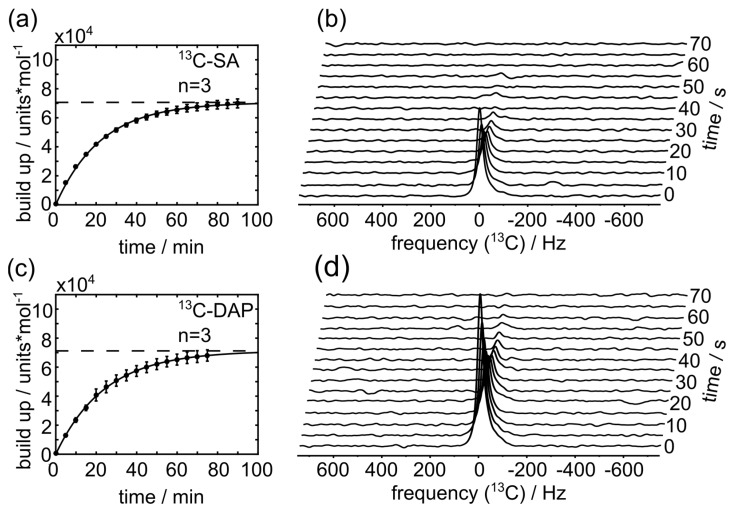
**Molar solid-state polarization build-up (a,c) and polarization decay in aqueous solution (b,d) of DAP and SA.** The maximum achievable molar solid-state polarization of DAP (**a**) and SA (**c**) were measured to 7.12 × 10^4^ u.*mol^−1^ and 7.06 × 10^4^ u.*mol^−1^, respectively (from fit, horizontal dashed lines). After dissolution, the decaying ^13^C NMR signal (**b**,**d**) was used to quantify *T*_1_ = 13.8 s ± 0.4 s and *T*_1_ = 18.8 s ± 2.0 s, for SA and DAP, respectively (*B*_0_ = 1 T). The solid build up constants for SA and DAP were 22.5 ± 1.8 min and 24.2 ± 4.1 min, respectively. The build up constant of PA was 15.2 ± 0.5 min. Measured concentrations of DAP and SA were 20.9 ± 1.7 mM (pH 5.3 ± 0.8, *n* = 3) and 28.5 ± <0.1 mM (pH 5.8 ± 0.4, *n* = 3), respectively.

^13^C-SA and ^13^C-DAP hydrochloride form a glass at concentrations up to 6 M and 7 M, respectively, and show a 30% smaller maximum solid state polarization compared to [1-13C]pyruvate (PA, Figure 4 and Figure S5). However, we observed that the HP signal of SA and DAP rapidly relaxed during the dissolution process in aqueous buffers yielding poor or no analyzable NMR spectra. Addition of vitamin C to the dissolution buffer allowed *T*_1_ measurements with concentrations relevant for in vivo applications (20–30 mM) in aqueous solution (Figure 4, Table S1 for vitamin C experiments) with reasonable liquid state polarization levels and signal enhancements *ε* (*n* = 3 measurements each): *P*(SA) = 9.5 ± 4.9%, *ε*(SA) = (1.1 ± 0.6) × 10^5^ and *P*(DAP) = 7.5 ± 2.1%, *ε* = (0.8 ± 0.2) × 10^5^ compared to *P*(PA) = 38.0 ± 1.4%. *T*_1_ values were 13.8 ± 0.4 s and 18.8 ± 2.0 s for SA and DAP, respectively (*T*_1_ (PA) = 62.3 ± 2.9 s). At pH values above neutral, the effect of vitamin C appeared to be weaker (Table S1). 

**Figure 5:**
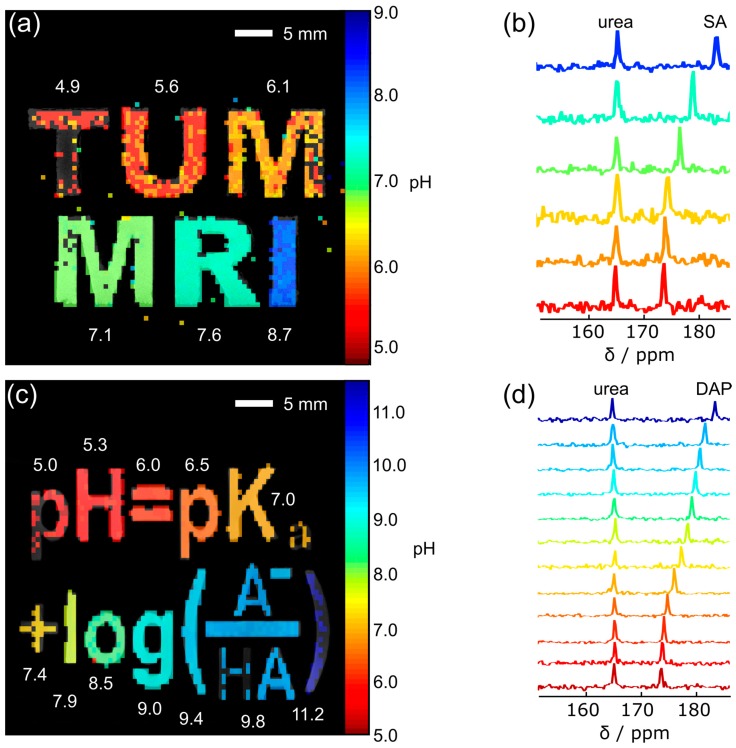
pH maps and ^13^C-NMR spectra of thermally polarized SA and DAP. 3D-printed, letter-shaped compartments filled with solutions containing 250 mM ^13^C-SA (**a**) or 250 mM ^13^C-DAP (**c**) both with 250 mM ^13^C-urea and 2 mM Dotarem at different pH. Chemical shift images were acquired and used to calculate pH maps ((**a**,**c**) color) co-registered with *T*_1_-weighted ^1^H images (gray). pH values measured with a pH electrode are written in white next to the respective compartment. NMR spectra of a representative voxel for each compartment are displayed for SA (**b**) and for DAP (**d**).

Finally, we acquired pH maps of thermally polarized, ^13^C-labelled SA and DAP model solutions in 3D-printed, letter shaped compartments. SA solutions of distinct pH values were used to image the abbreviation of “Technical University of Munich does Magnetic Resonance Imaging” (TUM MRI, Figure 5a). DAP solutions were used to image a phantom shaped to resemble the Henderson-Hasselbalch equation (pH = p*K*_a_ + log(A^−^/HA), Figure 5c). The low ^13^C signal at the edges of the setup (“T”, “M” in Figure 5a, “p”, “a,” and “)” in Figure 5c) may be attributed to the low *B*_1_ sensitivity of the coil in these regions (Figure S6). Nevertheless, pH values were recovered with a good accuracy except for those outside of the sensors range (pH < 6, Table S2). 

## 4. Discussion

Natural amino acids exhibit pH-dependent carbonyl carbon chemical shifts around the p*K*_a_ values of their carboxylic acid, their amino and their side chain groups. However, these p*K*_a_ values do not lie in the pH range that is useful for MR pH imaging, and the carbonyl chemical shift sensitivity of natural amino acids is small in that range (even for His and Cys). The physiological pH of blood and most bodily fluids is between pH 7.35 and pH 7.45 (up to pH 7.6), and diseases such as cancer exhibit an acidic extracellular tissue with pH as low as pH 5.8 [1,3,4,5,6]. The ideal p*K*_a_ of an in vivo pH sensor should therefore be around pH 7.0 ± 0.6 in order to achieve maximum pH sensitivity. Amidation or esterification of amino acids removes the carboxylic acid proton and the molecule thus loses the pH sensitivity of its carboxylic acid. In effect, this lowers the p*K*_a_ of the amino group toward pH values of physiological relevance. The addition of groups with positive inductive effects increases the p*K*_a_, which we demonstrated for basic and phosphorylated amino acids and that also applies for dicarboxylic acids, pyridine derivatives [27], and most probably for other pH-sensitive molecules like furanones [40]. On the other hand, addition of moieties or atoms with negative inductive effects (-I) decreases the p*K*_a_.

Spectroscopy and MRI (^1^H, ^19^F, ^31^P) with molecules bearing pH-sensitive chemical shifts are easily applicable approaches to measure pH in vivo*.* IEPA and ISUCA were used to measure pH in pre-clinical studies, and histidine could sense pH in human brain after oral loading. However, a main drawback of thermally polarized sensors is the requirement of high sensor doses and long scan times. In fact, concentrations were two [14,16,18] to three [15,17] magnitudes higher, and scan times about one (for single voxel spectroscopy) [15] to two magnitudes (imaging) [16,17] longer, compared to in vivo studies with hyperpolarized sensors [40]. 

Hyperpolarization enables MR imaging with up to more than 10^4^–10^5^ fold signal enhancements. This reduces scan times to a few seconds yielding MRSI images with high signal to noise ratios and a comparable resolution as obtained with thermally polarized sensors (IEPA: (2 × 2 × 4) mm^3^ [16], ^13^C-bicarbonate: (2 × 2 × 6) mm^3^ [3], nominal resolution). Furthermore, HP allows low sensor doses of ≤0.05 mM/kg [40], which potentially minimizes toxic side effects caused by the injection of highly concentrated exogenous compounds. Nevertheless, isotope enrichment and especially DNP instrumentation are rather expensive, whereas other HP techniques like PHIP can be implemented at lower costs. 

Spin-1/2 nuclei can be polarized using various polarization techniques. Carbon- and nitrogen-bearing compounds can be isotopically enriched with ^13^C and ^15^N in amino acid derivatives, and ^31^P is highly abundant in nature. Even though serine alkyl esters might form unhealthy alcohol degradation products, unsaturated precursors for pH sensors with a sensitivity up to Δδ = 4.5 ppm (e.g., serine allyl ester) could be synthesized which are potentially amenable for parahydrogen (H_2_) induced polarization (PHIP, Figure S7) [41]. In addition to ^13^C, amino acid derivatives (and histidine) bear pH-sensitive ^15^N and ^31^P nuclei. ^15^N could be a potential target for signal enhancement by reversible exchange (SABRE), which was already shown for imidazole—the side chain group of histidine [28]. This PHIP-based technique does not require chemical addition of H_2_ but has so far mainly been applied to cyclic, unsaturated, and most probably unhealthy pH-sensitive ^15^N-compounds [12]. Nevertheless, SABRE has already been used to enhance the proton NMR signal of amino acids, which could probably be extended to ^15^N [38]. Recently, ^31^P-phosphate and ^31^P-phosphocreatine have been successfully polarized with DNP, suggesting that phosphoserine and phosphothreonine could be polarized as well and could potentially be used for in vivo applications [29].

So far, two promising HP ^13^C-labelled chemical shift–based pH sensors for preclinical applications have been presented in the literature. ^13^C-diethyl malonic acid (DEMA) exhibits a long *T*_1_ (≈106 s, B_0_ = 11.7 T) and was used in vitro to sense pH [25], while [1,5-13C]zymonic acid (ZA) has already been applied for pH in vivo imaging in rodent kidneys and tumors [40,42]. Serine derivatives and DAP (Δδ = 4.2 ppm) exhibit a 2.4 or 1.5-fold higher pH sensitivity than diethyl malonic acid (Δδ = 1.7 ppm) and zymonic acid (Δδ = 2.4 ppm), respectively. ^13^C-SA and ^13^C-DAP appear to be biocompatible and they are stable in solution. Both molecules exhibit a reasonable solid state polarization level when polarized with DNP, but we observed a rapid signal loss after dissolution. This has been observed earlier for polarizations with 4-hydroxy-2,2,6,6-tetramethylpiperidine-1-oxyl (TEMPOL) radicals [43], for natural amino acids and molecules with amino groups directly attached to ^13^C-carbonyls (e.g., urea) [44]. Studies in absence and presence of vitamin C and at different pH values (Table S1) showed that vitamin C, which most likely scavenges [43] the radical, is essential to preserve hyperpolarization during the dissolution process or during transfer of the probe from the polarizer to the NMR spectrometer through transient Earth’s magnetic field. At pH values above neutral, the quenching effect of vitamin C was weaker, and, in dissolutions without vitamin C, relaxation effects were reduced. Liquid state polarization of SA and DAP could be further improved, e.g., by filtration (radical and paramagnetic impurities) and the usage of magnetic transfer lines [37,44,45,46,47,48,49,50,51,52,53]. Although the pH sensitivity of SA and DAP is higher than for diethyl malonic acid and zymonic acid, their *T*_1_ is relatively short (up to ≈18 s at B_0_ = 1 T) compared to DEMA (≈106 s, B_0_ = 11.7 T) [25] and ZA (≈56 s at B_0_ = 1 T) [42]. Deuteration, dissolution in D_2_O [33,42], or the use of long-lived singlet states [54,55,56] could enhance the HP signal lifetime and will be subject for further studies. 

Future in vivo application of pH sensitive amino acid derivatives will require their co-polarization with an internal reference without chemical shift sensitivity to back-calculate the pH. As reported earlier [40], ^13^C-urea is feasible, and we are seeking to establish recipes for co-polarization to provide optimal protocols for in vivo pH measurements. 

## 5. Conclusions

We systematically analyzed the effect of carbon chain length reduction and derivatization of naturally occurring amino acids on their pH-dependent chemical shift sensitivity. Thereby, we have found a strategy to tailor the p*K*_a_ of molecules toward the physiologically relevant pH range, which is necessary for the development of magnetic resonance pH biosensors. Notably, amino acid derivatives bear different spin-1/2 nuclei that exhibit a pH-dependent chemical shift in the physiological range and that can be polarized using DNP. Two novel pH sensors that can be potentially applied in vivo, namely [1-^13^C]serine amide and [1-^13^C]-2,3-diaminopropionic acid, have been identified. These molecules showed reasonable solid-state polarization levels and, to the best of our knowledge, exhibit the largest ^13^C chemical shift pH sensitivity known so far in the literature. Our findings elucidate the effect of chemical derivatization on MR pH sensitivity and are potentially transferable to other molecule classes.

## Figures and Tables

**Table 1:** Sample preparation scheme for hyperpolarization.

Compound	c (OX063)/mM	c (Dota)/mM	Solvent	c_final_/M	ν/GHz
SA	24	3	60% 10 M NaOH, 40% H_2_O	6	94.165
DAP	26	7	90% 10 M NaOH, 10% glyc	7	94.155
PA	16	1	self-glassing	14	94.172

OX063, trityl radical, Oxford Instruments (Abingdon, UK); Dota, Dotarem gadolinium chelate, Guerbet (Villepinte, France); glyc: glycerol.

**Table 2:** Fit parameters for back-calculation of pH maps for ^13^C-SA and ^13^C-DAP.

Sensor	p*K*_a1_	p*K*_a2_	*δ_min_*	*δ_max1_*	*δ_max2_*
^13^C-SA	7.35	-	172.44	180.67	-
^13^C-DAP	6.95	9.65	173.28	179.00	183.7

## Supplementary Materials

Supplementary information is available online at http://www.mdpi.com/1424-8220/18/2/600/s1. Figure S1: pH-dependent chemical shift of amino acids and derivatives; Figure S2: pH dependent chemical shifts of cysteine and histidine; Figure S3: Stability of amino acid esters in aqueous solution; Figure S4: ^13^C spectra from NMR titration series of Phosphoserine (P-Ser) and Phosphothreonine (P-Thr); Figure S5: Molar solid state polarization build ups of ^13^C-DAP, ^13^C-SA and [1-13C]pyruvate; Figure S6: B_1_-field homogeneity of the ^1^H/^13^C volume coil; Figure S7: Amino acid esters as potential targets for ^13^C parahydrogen induced polarization (PHIP). Table S1: Hyperpolarization experiments of ^13^C-DAP in absence and presence of vitamin C at varying pH; Table S2: Electrode and 13C-calculated pH values of spatially resolved pH measurements with letter shaped 3D printed phantoms.
