# Supplementary material for: Hyperpolarized Amino Acid Derivatives as Multivalent Magnetic Resonance pH Sensor Molecules
Source: Sensors (Basel). 2018 Feb 15;18(2):600. doi: 10.3390/s18020600 (PMC5856118; doi:10.3390/s18020600)
Supplement: Supplementary file 1 [file sensors-18-00600-s001.pdf]

Supplementary Information

Hyperpolarized amino acid derivatives as multivalent magnetic resonance pH sensor molecules

Christian Hundshammer [1,2,3], Stephan Düwel [1,2,3], David Ruseckas [2], Geoffrey Topping [1], Piotr Dzien [1], Christoph Müller [4,5,6], Benedikt Feuerecker [1,5], Jan. B. Hövener [7], Axel Haase [3], Markus Schwaiger [1], Steffen J. Glaser [2] and Franz Schilling [1,*]

[1] Department of Nuclear Medicine, Klinikum Rechts der Isar, Technical University of Munich, 81675 München, Germany; christian.hundshammer@tum.de (C.H.); stephan.duewel@tum.de (S.D.); geoff.topping@lrz.tu-muenchen.de (G.T.); realtoughmonkey1980@gmail.com (P.D.); benedikt.feuerecker@mytum.de (B.F.); markus.schwaiger@tum.de (M.S.)

[2] Department of Chemistry, Technical University of Munich, 85748 Garching, Germany; david.ruseckas@mnet-online.de (D.R.); glaser@tum.de (S.J.G.)

[3] Munich School of Bioengineering, Technical University of Munich, 85748 Garching, Germany; axel.haase@tum.de

[4] Department of Radiology, Medical Physics, University Medical Center Freiburg, Faculty of Medicine, University of Freiburg, 79106 Freiburg, Germany; christoph.mueller.rdiag@uniklinik-freiburg.de

[5] German Consortium for Cancer Research (DKTK), 69120 Heidelberg, Germany

[6] German Cancer Research Center (DKFZ), 69120 Heidelberg, Germany

[7] Section for Biomedical Imaging, Molecular Imaging North Competence Center (MOINCC), Department for Radiology and Neuroradiology, University Medical Center Kiel, University Kiel, 24118 Kiel, Germany; jan.hoevener@rad.uni-kiel.de (J.B.H.)

* Correspondence: fschilling@tum.de; Tel.: +49-(89)-4140-4586

Supplementary Figures 3
Figure S1: pH-dependent chemical shift of amino acids and derivatives 3
Figure S2: pH dependent chemical shifts of cysteine and histidine 4
Figure S3: Stability of amino acid esters in aqueous solution 5
Figure S4: ^{13}C spectra from NMR titration series of Phosphoserine (P-Ser) and Phosphothreonine (P-Thr) 6
Figure S5: Molar solid state polarization build ups of ^{13}C-DAP, ^{13}C-SA and [1-^{13}C]pyruvate 6
Figure S6: B_1-field homogeneity of the ^{1}H/^{13}C volume coil 7
Figure S7: Amino acid esters as potential targets for ^{13}C parahydrogen induced polarization (PHIP) 8
Supplementary Tables 9
Table S1: Hyperpolarization experiments of ^{13}C-DAP in absence and presence of vitamin C at varying pH 9
Table S2: Electrode and ^{13}C-calculated pH values of spatially resolved pH measurements with letter shaped 3D printed phantoms 10
References 11

Supplementary Figures

Figure S1: pH-dependent chemical shift of amino acids and derivatives

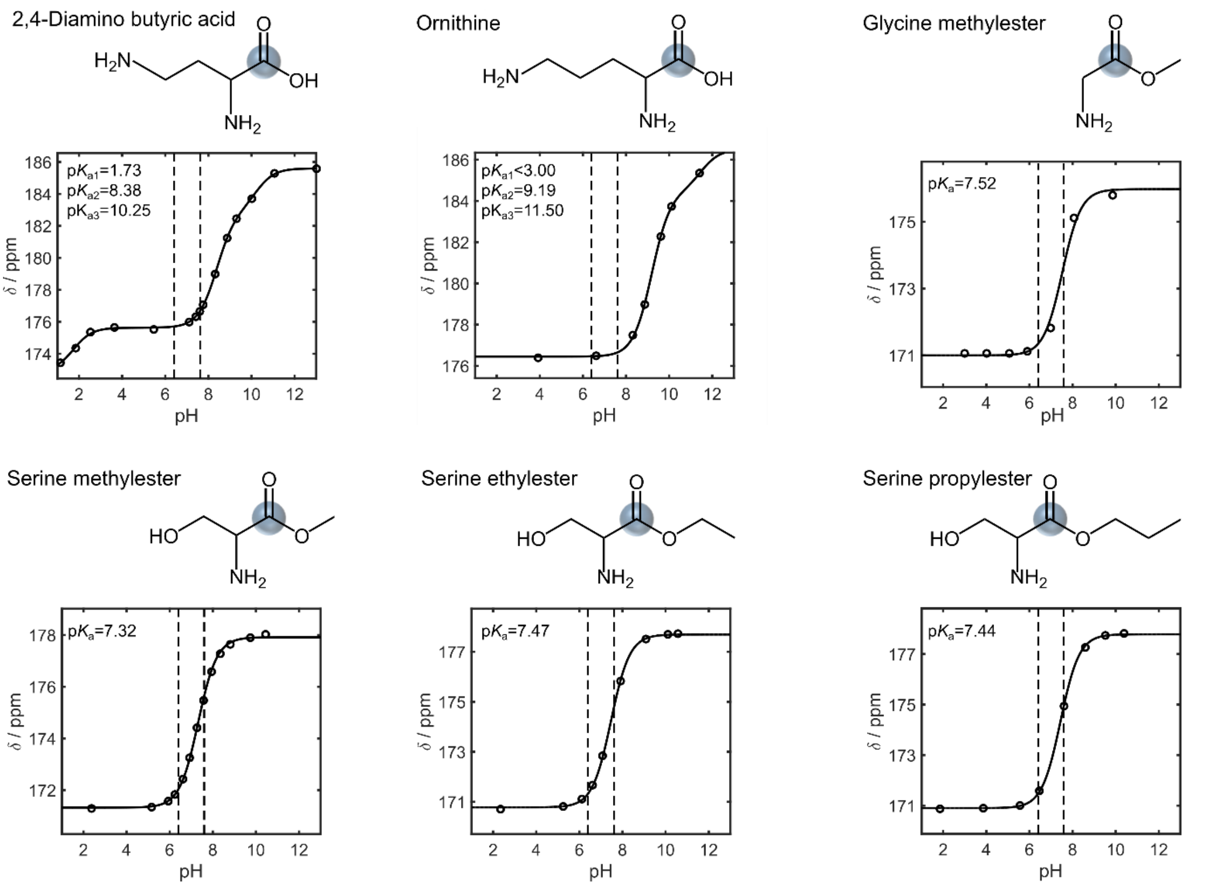

Figure S1: pH dependent chemical shift of carbonyl ^{13}C-atoms of 2,4-diamino butyric acid, ornithine, glycine methyl ester, serine methyl, ethyl and propyl ester. Chemical structures of the respective amino acids, NMR titration curves and pK_a values calculated from the fit are given. The pH range relevant for pH *in vivo* imaging is indicated with vertical dashed lines in each pH NMR titration plot.

Figure S2: pH dependent chemical shifts of cysteine and histidine

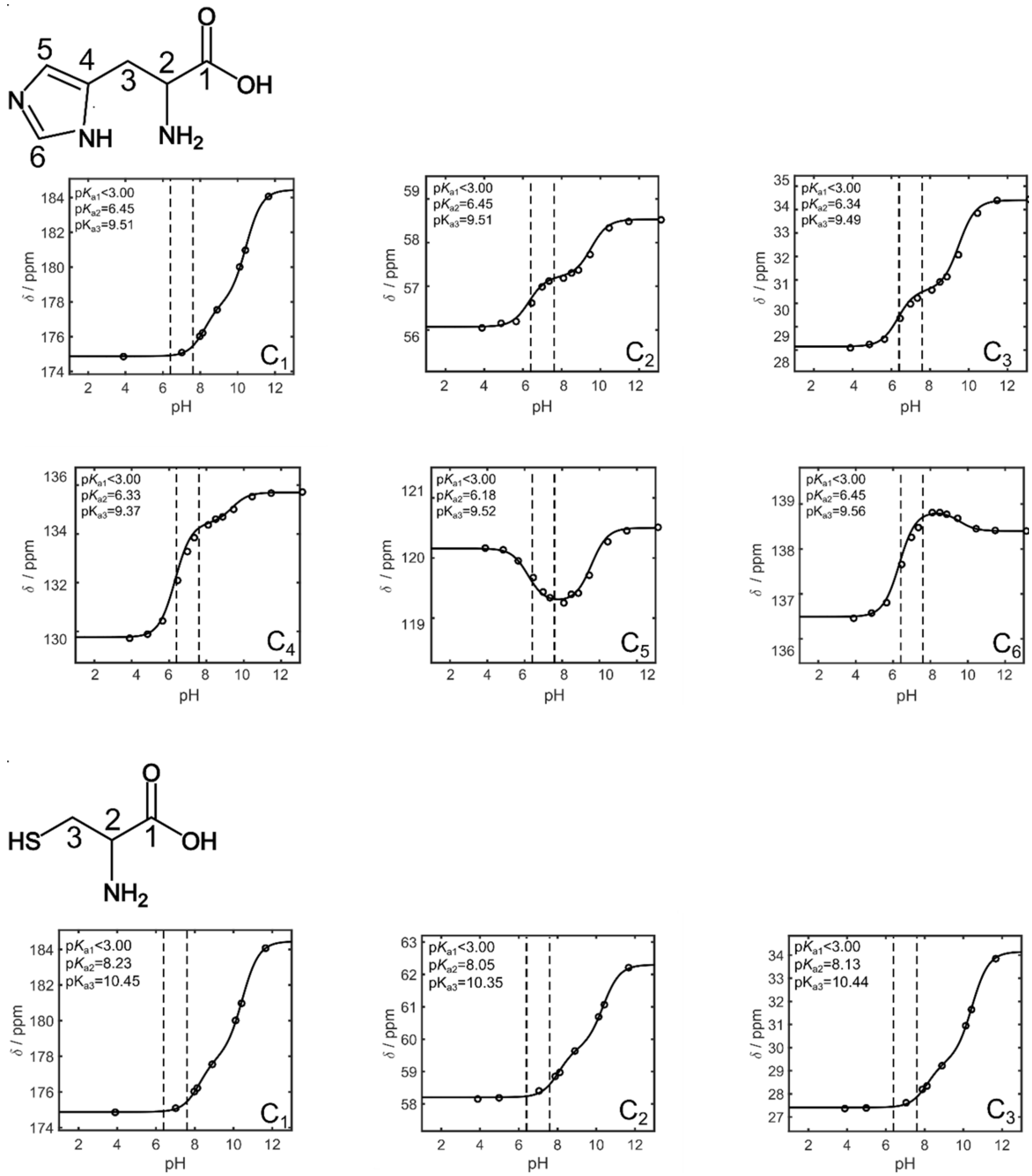

Figure S2: NMR pH titration curves of histidine (top) and cysteine (bottom). Chemical structures of both amino acids and the respective pH dependent chemical shifts are given. Histidine and cysteine bear side chains groups with a pK_a close to a range that is relevant for pH *in vivo* imaging. However, their chemical shift sensitivity between pH 6.4 and pH 7.6 of all carbon atoms is rather low.

Figure S3: Stability of amino acid esters in aqueous solution

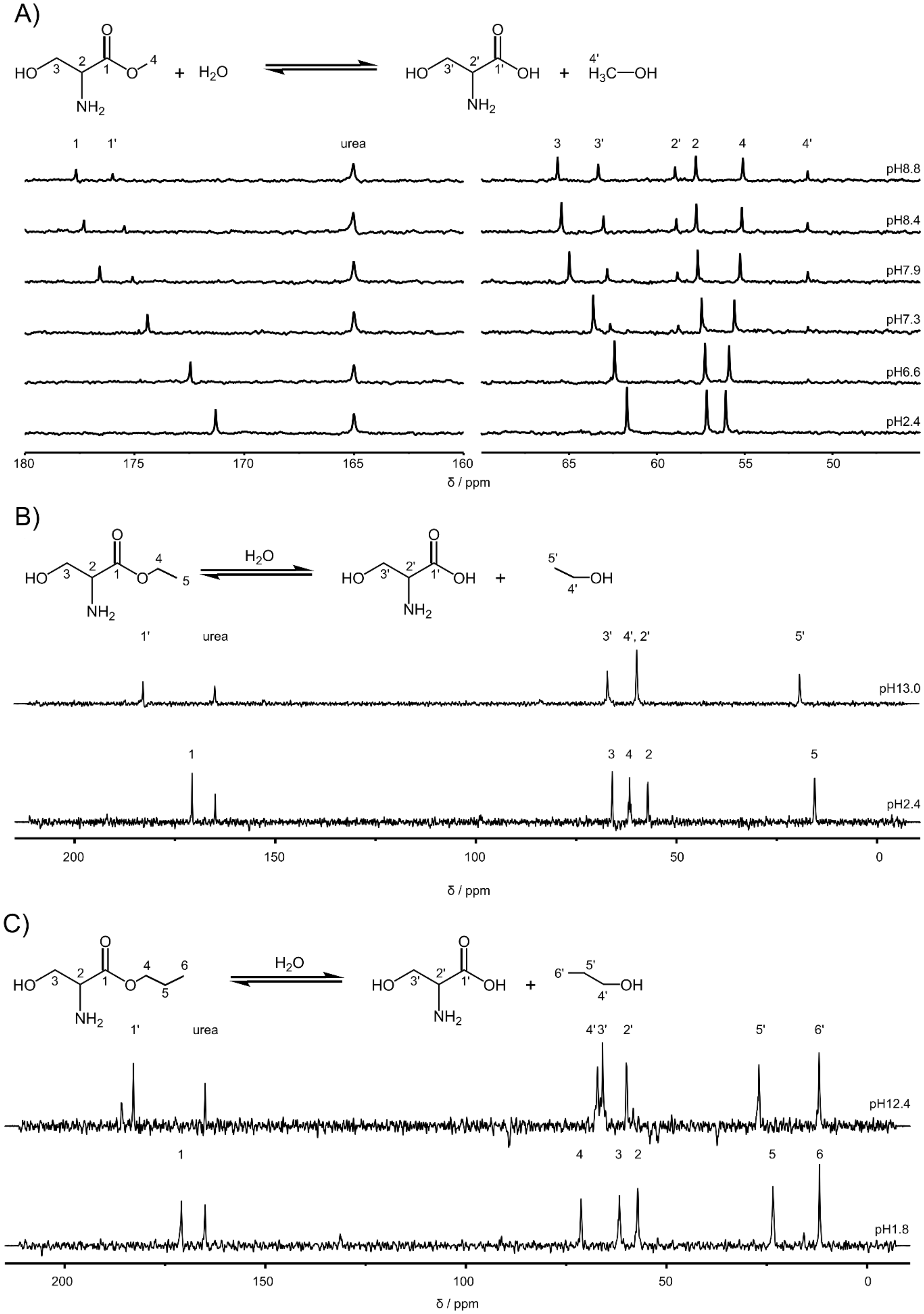

Figure S3: Stability of serine alkyl esters in aqueous solution. **A**) NMR titration series of serine methyl ester shows that the molecule hydrolysis in aqueous solution forming serine and methanol. **B**) First and last spectrum of an NMR titration series of serine ethyl ester. The amino acid derivative is stable at low pH and starts hydrolyzing at pH>10. The NMR titration series took less than 1 hour. **C**) The same observation as in **B**) was made for serine propyl ester.

Figure S4: ^{13}C spectra from NMR titration series of Phosphoserine (P-Ser) and Phosphothreonine (P-Thr)

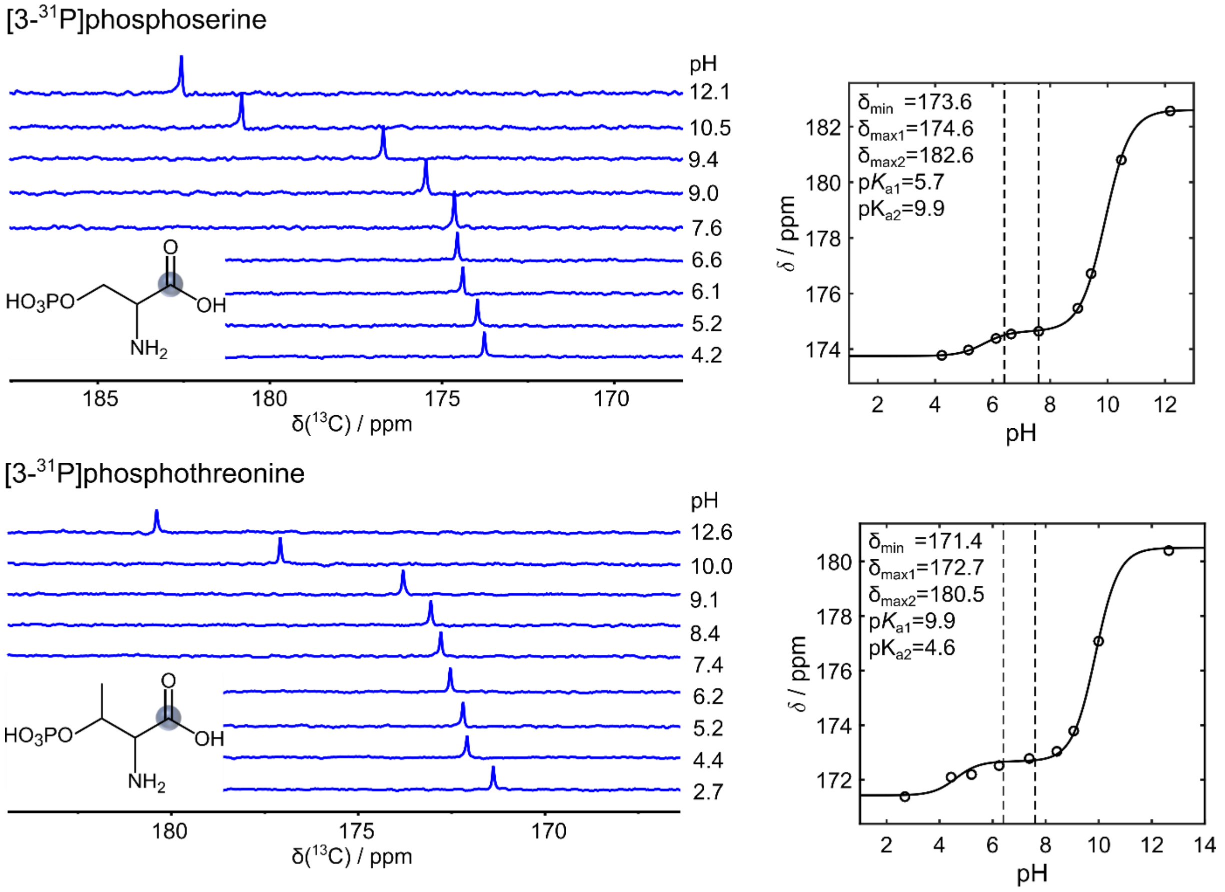

Figure S4: ^{13}C-NMR pH titration series of [3-^{31}P]phosphoserine (P-Ser) and [3-^{31}P]phosphothreonine (P-Thr). In **A**) and **C**) the chemical structure and the respective NMR signal of the carboxyl carbon atom (circled in blue in the structure) of P-Ser and P-Thr shifting with pH is shown. In **B**) and **D**), the pH-dependent chemical shifts are plotted against pH and fits are given with the respective fitting parameters. The pH range relevant for pH *in vivo* imaging is indicated with vertical dashed lines in each pH NMR titration plot.

Figure S5: Molar solid state polarization build ups of ^{13}C-DAP, ^{13}C-SA and [1-^{13}C]pyruvate

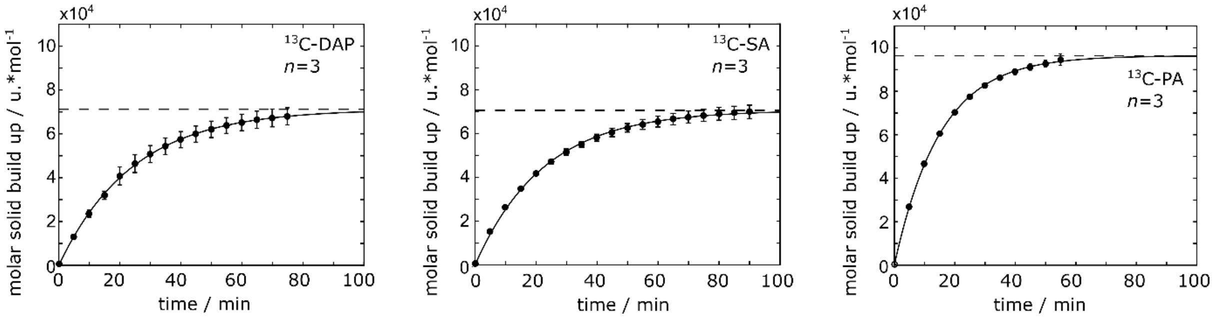

Figure S5: Molar solid state polarization build-up of DAP and SA compared and pyruvate. The maximum molar solid state polarization build-up of DAP and SA is about 30 % smaller than the one of pyruvate. The fitted maximum solid state polarization level is indicated by horizontal dashed lines. The solid build up constants for SA, DAP and PA were 22.5 ± 1.8 min, 24.2 ± 4.1 min and 15.2 ± 0.5 min, respectively.

Figure S6: B_1-field homogeneity of the $^1H/^{13}C$ volume coil

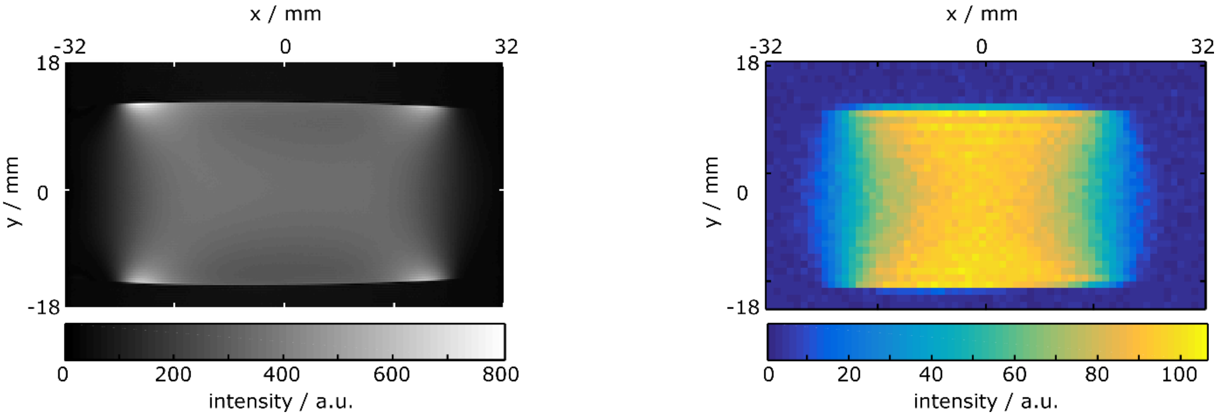

Figure S6: ^{13}C-Images of ^{13}C-urea in water uniformly filling the entire field of view of the $^1H/^{13}C$ volume coil. A) shows a coronal proton image. **B**) shows the ^{13}C signal in the same orientation as the proton image indicating a lower B_1 field away from the axial center of the $^1H/^{13}C$ volume coil.

The spatially varying sensitivity of the $^1H/^{13}C$ volume coil was assessed for ^{13}C-CSI by acquiring an image of a uniform phantom containing 1.8 M ^{13}C-urea and 50 mM DOTA, with diameter 28.7 mm (50 mL Falcon tube), and length extending past both axial ends of the sensitive volume of the coil. A horizontal image was acquired with a 2D phase-encoded chemical shift imaging sequence, with repetition time 35 ms, flip angle 60°, matrix size 64x36, field of view (64x36) mm^2, slice thickness 2 mm, 9 averages, total scan time 2 min 6 s, receive bandwidth 2 kHz, 64 points acquired per phase-encode, spectral resolution 5.6 Hz, excitation bandwidth 12 kHz, and chemical shift offset 163.5 ppm. Automated linear shimming on the proton signal from the phantom was run prior to ^{13}C-image acquisition. ^{13}C-images were reconstructed in Matlab, and then averaged over frequencies within +/- 50 Hz of the peak frequency at the centre of the phantom. The reconstructed sensitivity image covers the entire sensitive length and diameter of the volume coil, and the uniform phantom completely fills the sensitive volume.

The cause of intensity variation is the variation of transmit and receive B_1 of the coil with position. Within the axially central 20 mm, the sensitivity is relatively uniform, but further away from the center of the coil, the sensitivity drops substantially. The relationship between phantom T_1, scan repetition time and flip angle, and coil B_1-variation is more complicated than warrants a full discussion here. However, this image illustrates the cause - coil B_1 variation - of reduced image intensity in areas further away from the axial center of the letter shaped phantoms.

Figure S7: Amino acid esters as potential targets for ^{13}C parahydrogen induced polarization (PHIP)

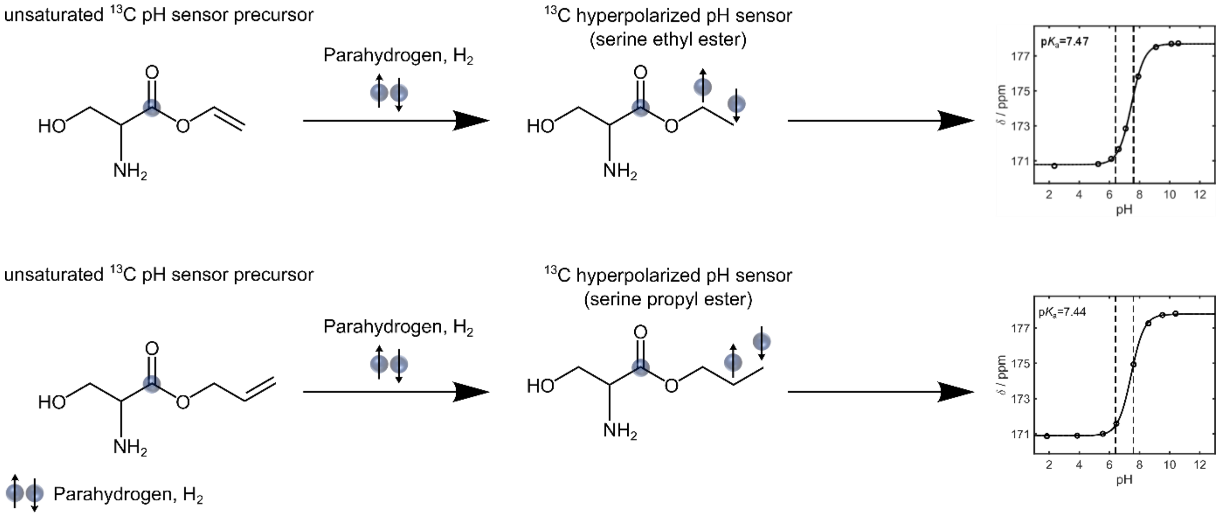

Figure S7: Amino acid esters as potential targets for ^{13}C parahydrogen induced polarization (PHIP). Unsaturated vinyl and allyl esters of serine are potentially amendable for chemical addition of parahydrogen yielding serine ethyl and propyl ester that are sensitive to pH at the physiological range.

The carbonyl carbon of pyruvate and acetate esters with unsaturated alkyl chains can be hyperpolarized by addition of parahydrogen.[1] Analogously, parahydrogen addition to unsaturated precursors of serine ethyl ester and serine propyl ester should yield the desired hyperpolarized pH sensors. Synthesis of amino acid allyl and vinyl esters has been described previously.[2,3]

Supplementary Tables

Table S1: Hyperpolarization experiments of ^{13}C-DAP in absence and presence of vitamin C at varying pH

Table S1: Hyperpolarization experiments of ^{13}C-DAP in absence and presence of vitamin C at varying pH.

				**Vitamin C**			
pH	4.08	5.45	5.59	6.20	7.24	7.99	8.8
Pol. Level / %	11.99	8.99	5.23	7.49	0.08	3.52	0.19
T1 / s	20.5	21.2	17.5	17.8	Low signal	22.2	21.3
Time to polarizer / s	29	21	22	22	22	22	26
				**No Vitamin C**			
pH	2.67	5.45	6.54	6.84 (n=2)	7.18	7.44	7.84
Pol. Level / %	No signal	No signal	No signal	No signal	No signal	No signal	0.32
T1 / s	No signal	No signal	No signal	No signal	No signal	No signal	17.1
Time to polarizer / s	25	24	25	24.5±0.7	23	27	22

Table S2: Electrode and ^{13}C-calculated pH values of spatially resolved pH measurements with letter shaped 3D printed phantoms

Table S2: Electrode and ^{13}C-calculated pH values of spatially resolved pH measurements with letter shaped 3D printed phantoms

Letter	pH image*	Electrode pH (± 0.01 std.)	Letter	pH image*	Electrode pH (± 0.01 std.)
T	5.71 ± 0.17	4.88	p	5.62 ± 0.23	5.00
U	5.80 ± 0.26	5.62	H	5.7 ± 0.1	5.30
M	6.07 ± 0.28	6.11	=	6.0 ± 0.1	6.00
M	6.97 ± 0.12	7.06	p	6.4 ± 0.1	6.50
R	7.40 ± 0.11	7.56	K_a	6.9 ± 0.04	7.01
I	8.93 ± 0.24	8.70	+	7.2 ± 0.04	7.41
			l	7.7 ± 0.05	7.85
			o	8.4 ± 0.30	8.50
			g	9.0 ± 0.04	9.00
			(	9.4 ± 0.04	9.32
			A^- / HA	9.8 ± 0.07	9.81
			)	11.3 ± 0.13	11.22

*pH values calculated from the pH images are mean±std of all voxels from the respective letter excluding signals that were 20 % smaller than the maximum intensity signal.

References

1. Reineri, F.; Boi, T.; Aime, S. Para-Hydrogen Induced Polarization of ^{13}C Carboxylate Resonance in Acetate and Pyruvate. *Nat. Commun.* **2015,** *6*, 5858.
2. Geckeler, K.; Bayer, E. Synthese von Aminosäure-Alkenylestern. *Chemische Berichte* **1974,** *107*, 1271–1274.
3. Friedrich-Bochnitschek, S.; Waldmann, H.; Kunz, H. Allyl Esters as Carboxy Protecting Groups in the Synthesis of O-Glycopeptides. *J. Org. Chem.* **1989,** *54*, 751–756.
